# I WISH I WAS DEAD: HEALTH CONSEQUENCES OF PHYSICALLY TRAUMATIZED OLDER ADULTS: A QUALITATIVE STUDY IN SOUTHERN GHANA

**DOI:** 10.1093/geroni/igad104.2907

**Published:** 2023-12-21

**Authors:** Abigail Appiah, Felix Apiribu, Veronica Dzomeku, Abigail Diji, Lilian Kolbila, Elizabeth Osei, Thomas Agyarko Poku, Peter Donkor

**Affiliations:** Suntreso Government Hospital (Ghana Health Service, Kumasi, Ashanti, Ghana; KNUST, Kumasi, Ashanti, Ghana; KNUST, Kumasi, Ashanti, Ghana; Kwame Nkrumah University of Science and Technology, Kumasi, Ashanti, Ghana; Ghana Health Service, Kumasi, Ashanti, Ghana; Ministry of Health, Accra, Greater Accra, Ghana; Ghana Health Service ( Suntreso Government Hospital), Kumasi, Ashanti, Ghana; KNUST, Kumasi, Ashanti, Ghana

## Abstract

The transformation from a physically healthy to a physically impaired older adults due to the effects of ageing, disability or injury can be a difficult transition to navigate. Comparatively, research on trauma in the older adult population is less as opposed to the younger adults. Ghana do not have appropriate policies and programs to support the growing numbers of the older adults. This indicates that the prevalence of the hidden problem of abuse will continue to increase, and create major public health problems. For this reason the study seek to explore the physical health consequences of the physically traumatized older adults. The study employed a descriptive phenomenological design and was guided by the Social-Ecological model. A purposive sampling technique was used to recruit participants for the study. A semi-structured interview guide was used to conduct individual in-depth face-to-face interviews. All interviews were audio-taped and transcribed verbatim. Data collection and analysis were done concurrently and data saturation was achieved after interviewing the eighth participant. Data were analyzed manually using thematic analysis and five (5) themes were generated: Lack of self-support ( inability to perform simple tasks) , Lack of family and friends support (neglect) , Lack of community and government support (no policies and programs to empower them. Physically traumatized older adults experienced extreme physical, emotional, mental and psychological discomfort as a result of their inability to do the things they wish to do, the neglect by immediate family, friends and the community as a whole and poor unfriendly transportation system.

